# A Polymer Blend Electrolyte Based on CS with Enhanced Ion Transport and Electrochemical Properties for Electrical Double Layer Capacitor Applications

**DOI:** 10.3390/polym13060930

**Published:** 2021-03-17

**Authors:** Shujahadeen B. Aziz, Elham M. A. Dannoun, Muhamad H. Hamsan, Hewa O. Ghareeb, Muaffaq M. Nofal, Wrya O. Karim, Ahmad S. F. M. Asnawi, Jihad M. Hadi, Mohd Fakhrul Zamani Abdul Kadir

**Affiliations:** 1 Hameed Majid Advanced Polymeric Materials Research Lab., Department of Physics, College of Science, University of Sulaimani, Qlyasan Street, Sulaimani 46001, Iraq; 2 Department of Civil Engineering, College of Engineering, Komar University of Science and Technology, Sulaimani 46001, Iraq; 3 General Science Department, Woman Campus, Prince Sultan University, P.O. Box 66833, Riyadh 11586, Saudi Arabia; elhamdannoun1977@gmail.com; 4 Centre for Foundation Studies in Science, University of Malaya, Kuala Lumpur 50603, Malaysia; hafizhamsan93@gmail.com (M.H.H.); mfzkadir@um.edu.my (M.F.Z.A.K.); 5 Department of Chemistry, College of Science, University of Sulaimani, Qlyasan Street, Sulaimani 46001, Iraq; hewa.ghareeb@univsul.edu.iq (H.O.G.); wrya.karim@univsul.edu.iq (W.O.K.); 6 Department of Mathematics and General Sciences, Prince Sultan University, P.O. Box 66833, Riyadh 11586, Saudi Arabia; muaffaqnofal@gmail.com; 7 Chemical Engineering Section, Universiti Kuala Lumpur Malaysian Institute of Chemical & Bioengineering Technology (UniKL MICET), Alor Gajah, Malacca 78000, Malaysia; asyafiq.asnawi@s.unikl.edu.my; 8 Department of Medical Laboratory of Science, College of Health Sciences, University of Human Development, Sulaimani 46001, Iraq; jihad.chemist@gmail.com

**Keywords:** polymer blend, plasticizer, ion transport, EEC modeling, FTIR deconvolution, TNM and LSV study, EDLC device

## Abstract

The fabrication of energy storage EDLC in this work is achieved with the implementation of a conducting chitosan–methylcellulose–NH_4_NO_3_–glycerol polymer electrolyte system. The simple solution cast method has been used to prepare the electrolyte. The impedance of the samples was fitted with equivalent circuits to design the circuit diagram. The parameters associated with ion transport are well studied at various plasticizer concentrations. The FTIR investigation has been done on the films to detect the interaction that occurs among plasticizer and polymer electrolyte. To get more insights into ion transport parameters, the FTIR was deconvoluted. The transport properties achieved from both impedance and FTIR are discussed in detail. It was discovered that the transport parameter findings are in good agreement with both impedance and FTIR studies. A sample with high transport properties was characterized for ion dominancy and stability through the TNM and LSV investigations. The dominancy of ions in the electrolyte verified as the *t_ion_* of the electrolyte is established to be 0.933 whereas it is potentially stable up to 1.87 V. The rechargeability of the EDLC is steady up to 500 cycles. The internal resistance, energy density, and power density of the EDLC at the 1st cycle are 53 ohms, 6.97 Wh/kg, and 1941 W/kg, respectively.

## 1. Introduction

In the past few years, researchers have been deeply concerned about global issues regarding sustainable energy sources that are safe for the environment. The most prominent electrolyte used in batteries and supercapacitor industries is an electrolyte in the liquid form. A liquid electrolyte (LE) provides high ionic conductivity due to its low bulk resistance but has drawbacks like leakage of harmful gases or corrosive liquid [1]. Researchers are attracted to study solid polymer electrolytes (SPEs). They are easier to handle due to their free-standing properties compared to LEs. Rapid technology development is needed in this era due to massive commercial demands on various electrical devices with different shapes and sizes. These demands can be solved with the implementation of SPEs [2]. Several advantages of SPEs include them being lightweight, of excellent thermal stability, high flexibility, cheap, and easy handling [3,4]. 

Plastic production worldwide has been boosted from ~2 million tons in 1950 to 8.3 billion tons in 2015. This is approximately an increase of 76% and results in increasing waste production. This plastic waste is in the environment, landfills, and oceans [5]. This is the main reason that biopolymers are studied in order to find alternatives to current non-biodegradable polymers. The most commonly studied marine biopolymer is chitosan (CS). CS can be a good ionic conductor due to its chemical structure (existence of OH and NH2) [6]. Methylcellulose (MC) is another biopolymer with great potential to be used in polymer electrolyte applications. MC is extracted when alkali-based cellulose is treated with methyl chloride. Its basic structural units are joined by β-(1→4) glycosidic bond [7,8]. Polymer blend, a method used in this work, is a straightforward process where two or more polymers form a superior material [9]. This is because the functional groups of each polymer interact with each other and provide more pathways for ions. CS and MC possess functional groups with lone pair electrons which are beneficial in ionic transportations.

A lot of ionic sources are available for SPE applications such as sodium, lithium, magnesium, potassium, silver, copper, and ammonium salts. This work is focused on the use of ammonium salts to fabricated protonic (H^+^) energy devices. H^+^ can be obtained not only from ammonium salts but also from inorganic acids like sulfuric acid (H_2_SO_4_) and phosphoric acid (H_3_PO_4_). Reports on these acids show a chemical degradation which is not good for energy device applications [10,11]. Ammonium nitrate (NH_4_NO_3_) is chosen in this work owing to its stumpy lattice energy of 648.9 kJ mol^−1^. This value can be considered low compared to other ammonium salts, e.g., for ammonium acetate (NH_4_CH_3_CO_2_), ammonium sulfate ((NH_4_)_2_SO_4_, and ammonium phosphate ((NH_4_)_3_PO_4_), the values are 703.1, 1754.7, and 3334.0 kJ mol^−1^, respectively. In addition, lattice energy for ammonium bromide (NH_4_Br), ammonium chloride (NH_4_Cl) and ammonium fluoride (NH_4_F) is 682.0, 708.0 and 834.0 kJ mol^−1^, respectively [12,13]. A low lattice energy salt provides more free ions and all of these free ions can thus contribute to the energy storage process of the energy devices.

The process of energy storage for an electrochemical double-layer capacitor (EDLC) involves a non-Faradaic reaction which means that ions build a charge double-layer on the carbon-based electrodes [14]. It has been reported that EDLC has higher power density and easier fabrication methods compared to Faradaic capacitor or pseudocapacitor [15,16,17]. Activated carbon is the most commonly used active material in the electrodes of an EDLC [18,19,20]. Various activated carbons have been synthesized from different sources such as nori-based [21], tofu-based [22], dimocarpus longan-based [23], coconut shell-based [24], and many more. Unique properties of activated carbon such as excellent electron conductivity, reasonable price, easy handling, and good chemical stability, plus high surface area mark it a choice for EDLC applications [25]. In this effort, CS is blended with MC to form a polymer matrix. Ions are provided by the addition of NH_4_NO_3_ and glycerol. The electrolyte with the best performance will be chosen to be used in the EDLC fabrication.

## 2. Experimental Methods

### 2.1. Electrolyte Preparation

The preparation of polymer electrolytes was performed from the components of MC (4000 cP), CS (MW 310,000–375,000 g/mol), and low MW glycerol plasticizer. In a separate flask, CS:MC polymer blends were prepared by dissolving 30 wt.% MC and 70 wt.% of CS in 40 mL of 1% acetic acid at ambient temperature for about 3 h. The two solutions were then combined and stirred for further 2 h in order to obtain a homogeneous solution. To this solution, 40 wt.% of NH_4_NO_3_ was added. Plasticization of the resulting CS:MC polymer blend solutions was performed by adding different quantities of 14, 28, and 42 wt.% glycerol. The obtained samples were denoted as MCCSNH1, MCCSNH2, and MCCSNH3, correspondingly. The contents of these samples were poured into the plastic petri dishes to cast the fabricated films, which were subsequently dried at ambient temperature. Prior to characterizations, the films were further dried using silica gel desiccant.

### 2.2. Characterization Techniques

#### 2.2.1. FTIR and EIS Analyses

HIOKI 3531 Z Hi-tester was implemented to gain the impedance spectra of all samples over a frequency range f = 50 Hz–1000 kHz. Small pieces of 2 cm in diameter were cut from the films to prepare discs, which were placed between the two stainless steel (SS) electrodes underneath a spring pressure.

Moreover, Fourier transform infrared (FTIR) spectroscopy analysis was also carried out to investigate the interaction and complexation among the polymers, salt, and plasticizer. The FTIR analysis in this work was employed using a Spotlight 400 Perkin–Elmer spectrometer with 1-cm^−1^ resolution in the range of 500–4000 cm^−1^. To further support the ionic conductivity studies, the ionic transport parameters; number density (*n*), ionic mobility (*µ*), and diffusion coefficient (*D*) based on the percentage of free ions were identified. The deconvolution method via the Gaussian–Lorentzian function was used to extract overlapping peaks as well as to correct the baseline of the curves.

#### 2.2.2. Transference Number Analysis (TNM)

**Figure 1:**
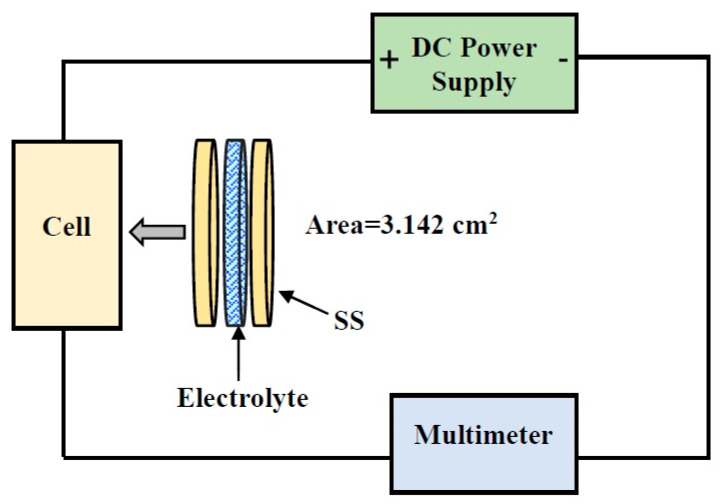
Circuit setup for Transference Number Analysis (TNM) measurement.

Transference number analysis (TNM) was conducted at a working voltage of 0.20 V using a V&A Instrument DP3003 digital DC power supply. The data were used to prove the involvement of ions in the entire conductivity. The electrolyte was sandwiched in between stainless steel (SS) electrodes. The summation of electronic (*t_e_*) and ionic (*t_i_*) transference number is equal to 1, where both values can be acquired from the subsequent equation:

(1) $$t_{i}=\frac{I_{i}-I_{ss}}{I_{i}}$$

where *I_ss_* and *I_i_* are the steady-state current and initial current, respectively. The circuit for TNM structure is shown in Figure 1.

#### 2.2.3. Linear Sweep Voltammetry (LSV)

The LSV analysis was performed using a Digi-IVY DY2300 potentiostat at a 0.02 V/s scan rate. The highest conducting sample was mounted in a Teflon holder with SS electrodes.

#### 2.2.4. Steps to Fabricate EDLC and Their Characterization

**Figure 2:**
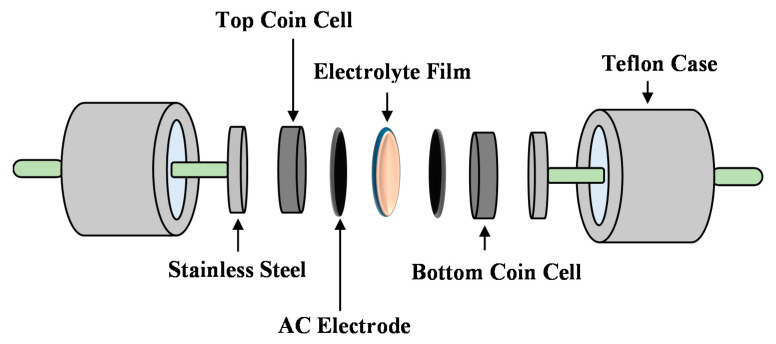
Illustration of the fabricated electrochemical double-layer capacitor (EDLC).

Mixing and pulverization of 0.25 g of carbon black (CB) and 3.25 g of activated carbon (AC) powders were carried out using a planetary ball miller (XQM-0.4) at a rotation speed of 50 rpm for 15 min. Meanwhile, polyvinylidene fluoride (PVDF) as a binder was stirred in 15 mL of N-methyl pyrrolidone (NMP) solvent. Dissolution of the AC-CB powder mixture in the NMP-PVDF solution resulted in the formation of a thick black solution, which then varnished on an Al-foil using a doctor blade. After oven drying of the coated foil at 60 °C, the dried electrodes were cut into circular samples with area of 2.01 cm^2^. The prime conducting electrolyte was situated between two carbon electrode CR2032 coin-type cells. As shown in Figure 2, these coin cells of EDLC were positioned in Teflon holders for further examination. The charge-discharge for the EDLC was measured using a Neware battery test system (current density = 0.5 mA/cm^2^). The equations below are allowed calculating each specific capacitance (C), equivalent series resistance (ESR), efficiency, energy density (E), and power density (P) of the EDLC.

(2) $$C_{}=\frac{i}{xm}$$

(3) $$ESR=\frac{V_{d}}{i_{}}$$

(4) $$efficiency=\frac{t_{dis}}{t_{cha}}\times 100\%$$

(5) $$E=\frac{CV}{\begin{array}{l} 2 \end{array}}$$

(6) $$P=\frac{V^{2}}{4mESR}$$

where *i* is the current apply, *x* the slope of discharge part, *V_d_* is the drop voltage and *m* stands for the mass of activate material used. The times for discharge and charge parts are given as *t_dis_* and *t_cha_*, respectively.

## 3. Results and Discussion

### 3.1. Impedance Study

**Figure 3:**
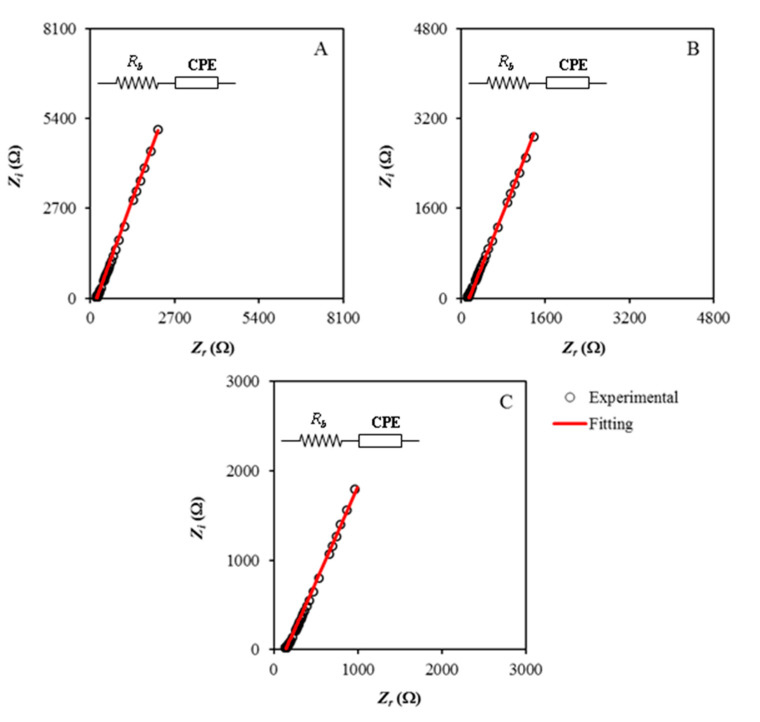
Nyquist plots of the electrolytes of (**A**) CMNG1, (**B**) CMNG2 and (**C**) CMNG3, respectively, at room temperature. The inset figure is the suitable electrical equivalent circuit.

PEs as part of an innovative class of materials are widely applied in devices [26]. Many investigational studies have confirmed the importance of adding low molecular weight plasticizers into the PEs for enhancing ion migration [27]. A significant role of impedance spectroscopy is the investigation of the electrical properties of a large variety of electrolyte materials and the electrode–electrolyte interfaces [28,29,30]. The impedance plots for the CS:MC:NH_4_NO_3_:glycerol system are depicted in Figure 3A–C. When the circuit composed of a capacitor and a resistor in parallel, the ideal impedance spectra should be a standard semicircle having a diameter-matched with the real axis. The absence of the high-frequency semicircular region indicates that the ions are primarily responsible for the conduction [31,32,33]. However, the presence of the spike at the low-frequency region results from the electric double-layer capacitance at the blocking electrodes [34,35,36,37]. As obviously seen in Figure 3A–C, the absence of the semicircles at the high-frequency region is owed to the use of the plasticizer [38]. This relation below is employed to calculate the impedance of CPE (*Z_CPE_*) [39,40]:

(7) $$Z_{CPE}=\frac{1}{C\omega^{p}}\left[\cos\left(\frac{\pi p}{2}\right)-i\sin\left(\frac{\pi p}{2}\right)\right]$$

where *C*, *ω* and *p* stand for the CPE capacitance, the angular frequency and the deviation measure of the plot from the axis, respectively. The CPE is connected in series with the *R_b_* of the electrolytes and their EIS signals only display the spike [41]. The impedance can be mathematically represented as follows [42]:

(8) $$Z_{r}=\frac{\cos\left(\frac{\pi p}{2}\right)}{C\omega^{p}}+R_{b}$$

(9) $$Z_{i}=\frac{\sin\left(\frac{\pi p}{2}\right)}{C\omega^{p}}$$

These equations are also used in the fitting parameters (CPE1 and CPE2) and determined the *R_b_* values precisely. The resulting *R_b_* values and CPE values for the CMNG1, CMNG2, and CMNG3 electrolytes are listed in Table 1. The high values of CPE2 with the addition of glycerol can be explained on the basis of increasing the number of ions in the electrolytes. This results in an increase in the accelerating electrode polarization; thereby, an increase in the capacitance value at low frequency [43]. The addition of glycerol as a plasticizer enhances salt dissociation and hence increases ion mobility [44,45].

From Equation (10), the ionic conductivity (*σ*) can be calculated as the measure of the electrical properties of the electrolytes. The calculation also comprises *R_b_*, *t*, and *A* values as charge resistance, thickness, and surface area, respectively. From Table 1, the conductivity values for the electrolytes are expressed by:

(10) $$\sigma =\frac{t}{A\times R_{b}}$$

Commonly the *σ_dc_* can be stated as follows [46]:

*σ_dc_* = Σ*q n µ* (11)

Previous investigations established that the H^+^ ion released by ammonium ion is considered as the charge carrier species in such polymer/ammonium salt systems [47]. The extra plasticizer amended the number of charge carriers (*n*) through further salt dissociation and increase mobility (*µ*) by providing huge amorphous phases. Some equations can be applied to calculate the transport parameters such as diffusion coefficient (*D*), mobility (*μ*), and number density (*n*) of ions from impedance data possessing only a spike [48]. Beginning with the calculation of the D of ionic species, the following equation is employed:

(12) $$D=D_{{}^{\circ}}\exp\{-0.0297{\left[\ln D_{{}^{\circ}}\right]}^{2}-1.4348\ln D_{{}^{\circ}}-14.504\}$$

(13) $$D_{{}^{\circ}}\;=\left(\frac{4k^{2}l^{2}}{R_{b}{}^{4}\omega_{\min}{}^{3}}\right)$$

where l is the electrolyte thickness, *ω_min_* is the angular frequency at the minimum *Z_i_* value.

The mobility (*µ*) of the ionic species is determined from Equation (14),

(14) $$\mu \;=\left(\frac{eD}{K_{b}T}\right)$$

where *T* is the absolute temperature and *k_b_* is the Boltzmann constant. 

Since DC conductivity of ions is obtainable from Equation (10), the density of ion carriers (*n*) can be determined from Equation (11). In Table 2, the parameters associated with the ion transport for all the systems are listed. There is a clear trend of increasing both values of *D* and *μ* with increasing glycerol quantity from 14 to 42 wt.%. These correlations are related to the chain flexibility enrichment in the presence of glycerol. The same trend of increasing *n* with increasing glycerol quantity is observed. All these, in turn, result in an increase in conductivity. A possible explanation of this is the role of glycerol molecules in enhancing the salt dissociation, thereby raising the number density of ion carriers [48]. In addition, the attractive forces between the anions and cations of the salt are greatly lowered [49]. Consequently, a higher number of NH_4_^+^ ions (*n_i_*) is created by NH_4_NO_3_ into the polymer.

### 3.2. FTIR Results

**Figure 4:**
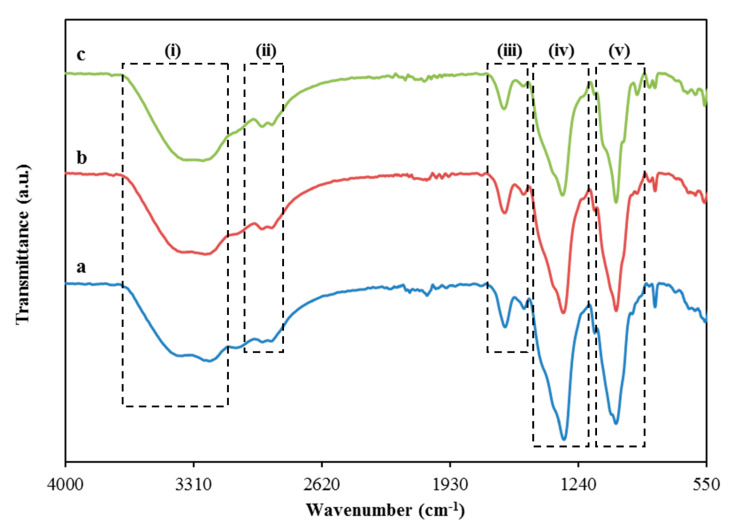
Fourier transform infrared spectroscopy (FTIR) spectra for (**a**) CMNG1, (**b**) CMNG2 and (**c**) CMNG3.

Figure 4 shows the FTIR spectra of the electrolytes at a wavenumber of 550–4000 cm^−1^. The bands due to -OH stretching and -OH bending can be found at (i) 3700–3150 cm^−1^ in a broad peak form and (iv) 1250–1500 cm^−1^ in a sharp peak form, respectively which caused by the addition of glycerol into the system [50,51]. Numerous peaks are observed in-band region (iv) where the one centered at 1332 cm^−1^ came from -OH bending. In addition, the observed peak ascribed to the NH_4_^+^ asymmetry elucidates a high tendency of the NH_4_^+^ distortion to release H^+^ ions [52]. Furthermore, the -CH symmetrical stretching and -CH asymmetrical stretching are found at the band region of (ii) 3050 to 2850c m^−1^ [53,54]. It is noticed that the intensity of the -CH bands is increased as the concentration of glycerol increases which signified the complex development between the glycerol and CS-MC-NH_4_NO_3_ [55]. The location carboxamide and amine band for the electrolytes are located at (iii) 1750–1520 cm^−1^ where a slight shift is observed when the concentration of glycerol increases. The shift shows the effect of glycerol concentration to provide more ions for the interaction with the oxygen and nitrogen atoms in the polymer blend [56]. Aziz et al. [57] and Shukur et al. [58] also reported a similar range of carboxamide and amine bands for their electrolyte systems. Lastly, the sharp peak located at (v) 900–1200 cm^−1^ possibly belongs to the C-O stretching which is comparable to the previous work reported by Mejenom et al. [59] and Poy et al. [60]. As the concentration of glycerol is increased, this band peak becomes sharper and causes a small shoulder peak to appear which also describes the interaction between the CS-MC-NH_4_NO_3_ and glycerol. 

**Figure 5:**
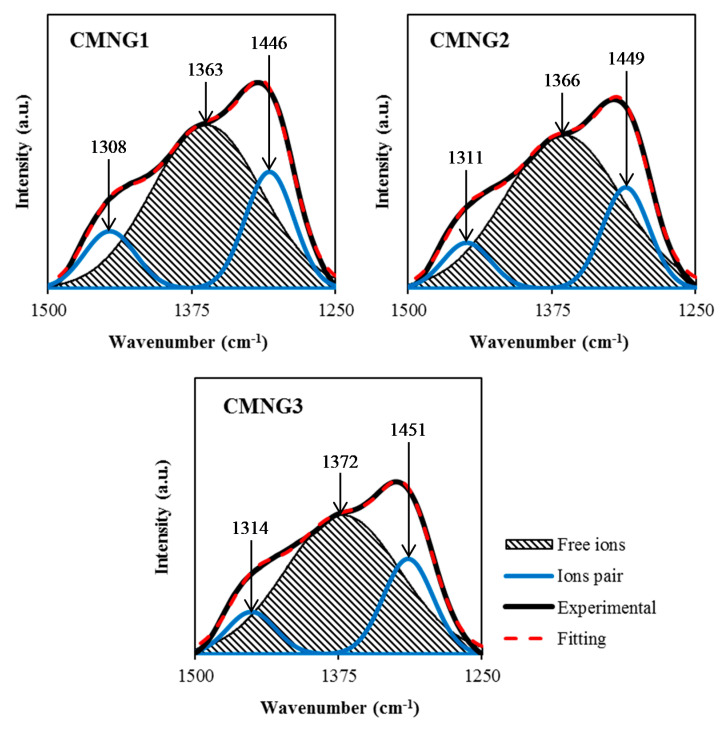
Deconvolution of FTIR spectra at 1250–1500 cm^−1^.

The FTIR deconvolution analysis is employed to support the ionic conductivity results where it also provides several advantageous properties such as resolving the overlapping peaks, removing noise and fringes from the spectrum, and inspecting the interconnection among deconvolution parameters [61]. Based on this approach, the deconvolution FTIR spectra can provide the fraction of conducting ions. The deconvolution possibility of FTIR spectra allowed one to isolate the existing peaks as well as alter both intensity and wavenumber as highlighted by Ramelli et al. [62]. Thus, the involvement of H^+^ in charge carriers can be verified. The opted band region for the deconvolution purpose was from 1250 to 1500 cm^−1^, as shown in Figure 5. This is payable to the evident interaction as before clarified in the FTIR analysis. In the same band region, the use of NH_4_NO_3_ led to shift the bands within the range of 1200–1500 cm^−1^, as reported by Kamarudin and Isa [63] and Hafiza and Isa [64]. Thus, this approach is intended to isolate the free and ion pairs appropriately. The free ions correspond to the free mobile ions like H^+^ while the ion pairs correspond to the ions of NH_4_^+^ or NO_3_^−^ [65,66,67]. With the help of the deconvoluted FTIR spectra shown in Figure 5, the percentage of free ions can be quantified using the following equation:

(15) $$\mathrm{Free}\;\mathrm{ions}\;\%=\frac{\mathrm{Area}\;\mathrm{of}\;\mathrm{free}\;\mathrm{ions}\;\mathrm{peak}}{\mathrm{Total}\;\mathrm{area}\;\left(\mathrm{free}\;\mathrm{ions}\;\mathrm{peak}+\mathrm{ions}\;\mathrm{pair}\;\mathrm{peak}\right)}\times 100$$

The percentage of free ions obtained by CMNG1 is 67.5%, which then significantly increased to 71.7% with 28 wt.% of glycerol. However, a slight decrease of the free ions percentage to 70.2% is observed when the concentration of glycerol is increased to 42 wt.%. This is generally explained that the percentage of free ions is influenced by the concentration of glycerol in the system which is highly correlated to the trend of the ionic conductivity. The increase of H^+^ ions that dissociated from NH_4_^+^ could also be a factor for the increment of ionic conductivity. Aniskari et al. [68] also reported the highest conducting electrolyte also had the highest percentage of free ions. By employing the percentage of free ions for each electrolyte, the transport parameters of number density (*n*), ionic mobility (*µ*), and diffusion coefficient (*D*), can be calculated using the Equations (16)–(18), where *N_A_* is Avogadro’s constant, *M* represents the number of moles of glycerol, and *e* is the elementary charge. *V_Total_* is the total volume of the polymer electrolytes.

(16) $$n=\frac{M\times N_{A}}{V_{Total}}\times \left(free\;ion\;\%\right)$$

(17) $$\mu =\frac{\sigma }{ne}$$

(18) $$D=\frac{\mu kT}{e}$$

The calculated transport parameters of the electrolytes are listed in Table 3 using FTIR routine. It can be observed that the trend of number density is followed the ionic conductivity pattern as in Table 1. The dissociation of H^+^ ions assists to facilitate the coordination of the H^+^ ions in the electrolyte system which leads to the enhancement of ionic conductivity value [69,70]. This can be further proved through the trend of *µ* and *D*. The flexibility and the segmental wave of the polymer chains in the system also predisposed the transport properties as well as the ionic conductivity [71]. However, the improvement of transport properties was observed when glycerol concentration reached 42 wt.% which is due to the enhancement of the chain flexibility that leads to the concentration increment of H^+^ carriers, and thus increases the ionic conductivity of the electrolyte.

### 3.3. Transference Number Study

**Figure 6:**
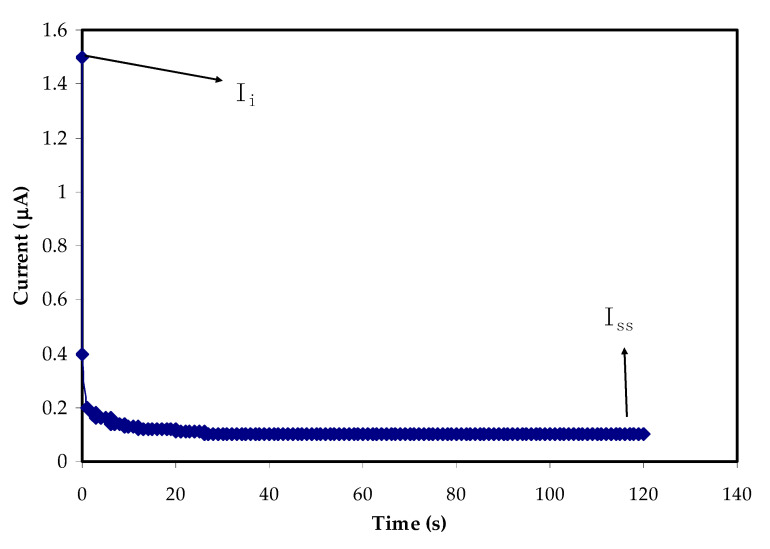
Polarization of the highest conducting electrolyte at 0.2 V.

The transfer of electrons and ions in an electrolyte provides conductivity. In order to determine the primary charge carrier, transference number (TNM) was evaluated at an applied voltage of 0.2 V. Figure 6 depicts polarization of the highest conductivity for the CS:MC:NH_4_NO_3_:glycerol system. It is obvious that at the beginning of the polarization process, the conduction of ions and electrons results in a significant current of 1.5 µA. As already mentioned, the evaluation is based on the SS electrode, owing to blocking the ions and permitting the electrons to pass. Therefore, as the time increases, the current minimizes up to a plateau at 0.1 µA. This steady-state current indicates the occurrence of a polarization process of the electrolyte. Thus, cations and anions are adsorbed at the negative electrode and the positive electrode, respectively [72,73]. At this stage, the current is only carried by electrons. The *t_e_* value of 0.07 and the *t_i_* value of 0.93 were obtained. Since *t_i_* is higher than *t_e_*, the dominant charge carriers of CS:MC:NH_4_NO_3_:glycerol electrolyte are the ions [74]. The accumulation of these ions on the surface electrodes allows EDLC applications. For the ammonium salt-based SPE reported by Vijaya et al. [75], the *t_i_* values were in the range of 0.90 to 0.99. Samsi et al. [76] obtained the *t_i_* value of 0.916 for cellulose acetate (75 wt.%)-NH_4_I (25 wt.%) system. In another work, the ions are also dominated (i.e., 0.85 out of 1) in ammonium based-poly(ethyl methacrylate) system [77]. Alia et al. [78] revealed the *t_i_* value of 0.905 for SPE of proton-based biopolymer with polycarbonate electrolyte as the plasticizer and zinc sulfate as the filler.

### 3.4. LSV Study

**Figure 7:**
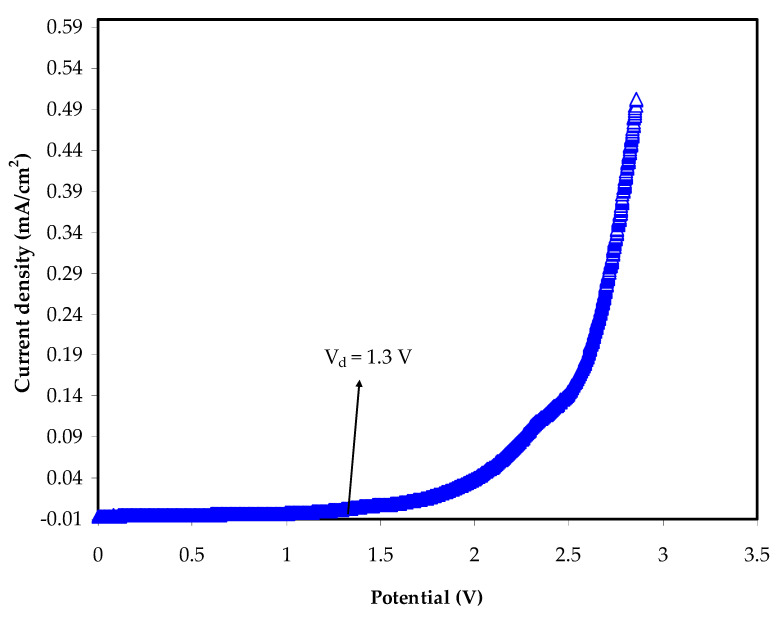
Linear Sweep Voltammetry (LSV) graph of the highest conducting electrolyte at 0.02 V/s.

EDLC is known as a high cycle supercapacitor, thus it is crucial to investigate the working voltage range, frequently referred to as potential stability [79]. The study employed LSV, facilitated by SS electrodes, to identify the potential stability of every polymer electrolyte, and Figure 7 illustrates a normal LSV graph for the highest conducting CS:MC:NH_4_NO_3_:glycerol blend electrolyte. The finding indicates that, from the EDLC application viewpoint [80], the potential stability is in the satisfactory working voltage range of 1.87 V. According to Monisha et al. [81], the threshold voltage is that the current passes through the cells. The potential stability obtained for this work is comparable to other reports with ammonium salts as the ionic provider. A complexation of ethylene carbonate, NH_4_NO_3_, and CS yield potential stability of 1.8 V [82]. A system of MC:NH_4_Br is reported to decompose beyond 1.53 V [19]. In another report, poly (ε-caprolactone) (PCL)-based polymer electrolytes are stable up to 1.4 V as ammonium thiocyanate is added [83]. 

### 3.5. Charge-Discharge Study

**Figure 8:**
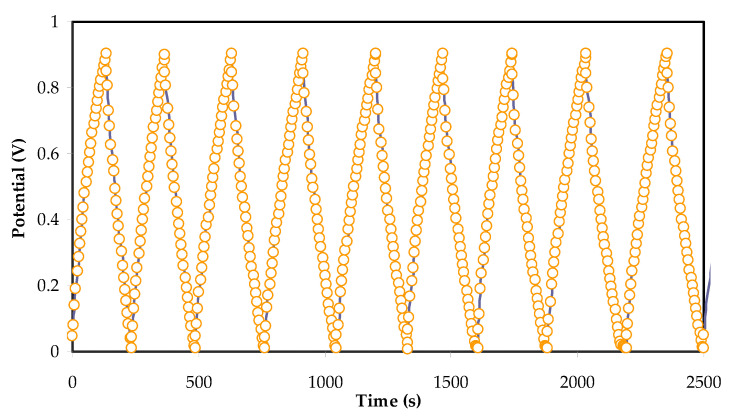
Selected charge–discharge graphs of the EDLC at 0.5 mA/cm^2^.

**Figure 9:**
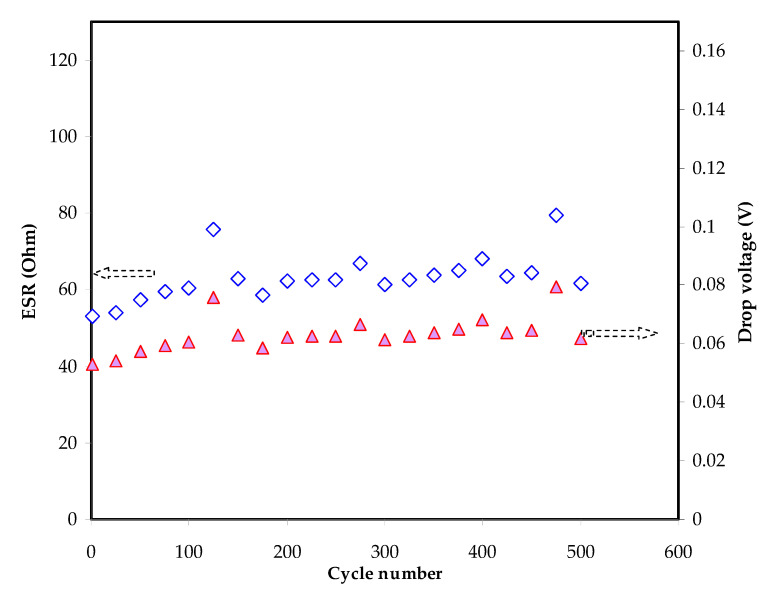
Equivalent series resistance (ESR) and drop voltage of the EDLC for 500 cycles of charging and discharging.

Figure 8 shows the charge-discharge cycle of the fabricated EDLC. An ideal capacitor will show a triangle curve due to polarization. Unlike conventional batteries where the discharge curve is non-linear due to the process of inter-and de-intercalation. Figure 9 shows the *V_d_* and ESR of the EDLC in this work. *V_d_* is energy loss and highly dependent on the internal resistance. *V_d_* can be observed before every discharging process where the calculated average value is 0.0631 V. The value of *V_d_* throughout the 500 cycles is quite stable at 125th and 475th cycles. This could be due to changes in ionic transportation pattern and the formation of the charge-double layer is disturbed. In addition to that, the ESR is also stable up to 500 cycles with an average of 63.1 ohms. A low value of ESR indicates that the constituents of EDLC have an excellent contact.

**Figure 10:**
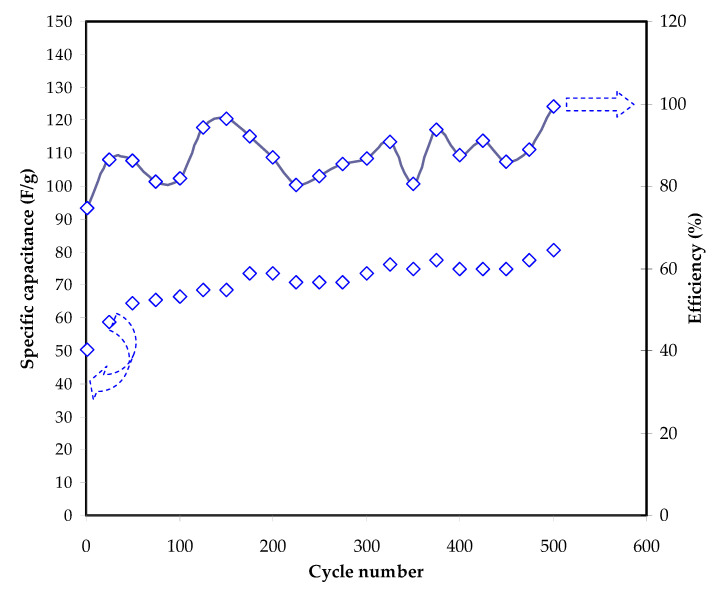
*C_s_* and efficiency of the EDLC against the cycle number at 0.5 mA/cm^2^.

Figure 10 illustrates the *C_s_* and efficiency as a function of cycle number for the fabricated EDLC at 0.5 mA/cm^2^. The *C_s_* value increases from 50.2 F/g at the 1st cycle to 68 F/g at the 150th cycle, followed by almost unchanging value after the 175th cycle upwards and reached an average value of 75 F/g when 500 cycles are completed. A rapid build-up of the double layers in EDLCs/supercapacitors is a main reason that the capacitance value is relatively low at the 1st cycle.

Negatively and positively charged ions are still employed to get used to the conduction pattern at the initial stage [84,85]. Table 4 shows an example of reported works related to the application of EDLC using various types of polymer electrolytes and materials. Many aspects have to be considered such as salts, polymer, filler, plasticizer, and electrodes used. 

The efficiency of the EDLC is illustrated in Figure 10. The high value of efficiency signifies that the discharging part is equal to or longer than the charging time. The efficiency of 74% can be observed at the first cycle. Usually, the charging process lasts longer in comparison with discharging. By subjecting the current, cations and anions begin approaching the electrode and become familiar with the conduction trend. A low efficiency is given at this stage. The efficiency is varied in the range of 80–99%. These findings of the present work reveal that the EDLC possesses good cycling stability and contact as well as less charge drop up to 500 cycles.

**Figure 11:**
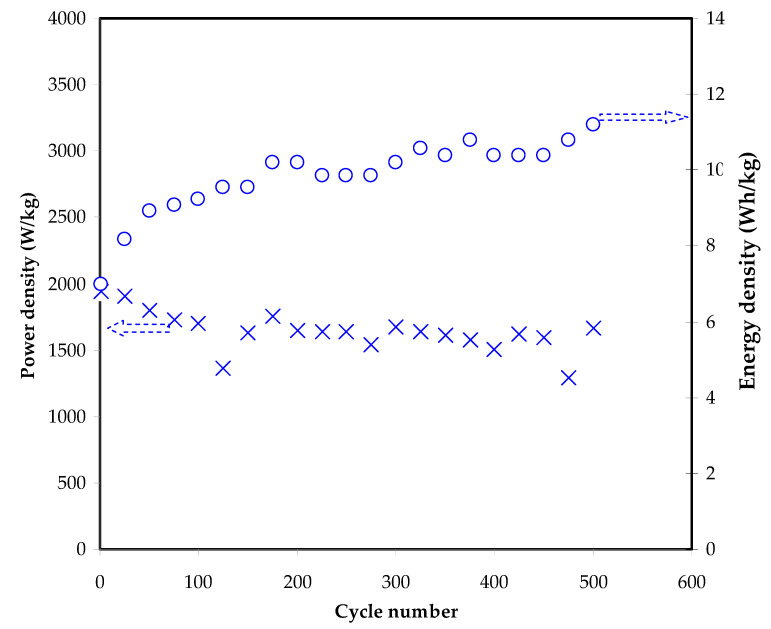
The plot of *E* and *P* of the EDLC versus the cycle number at 0.5 mA/cm^2^.

Figure 11 depicts the variation of *E* and *P* over 500 cycles, respectively. The *E* value was found to be 6.79 Wh/kg at the first cycle and then reached 9.52 Wh/kg. Beyond 175 cycles, E is clearly started to stabilize at an average value of 10.38 Wh/kg all over the 500 charge–discharge cycles. This fact clarifies that both NO_3_^−^ anions and NH_4_^+^ or H^+^ cations in the polymer chains of the CS:MC blend move towards the electrode surface at an almost similar energy barrier [90]. Winie et al. [91], demonstrated that a CS-based EDLC has energy density from 0.57 to 2.8 Wh/kg as the current density changes from 2 to 0.6 mA/cm^2^, respectively. The low ion aggregation is due to stability in energy density and capacitance pattern. The EDLC shows a higher power density than batteries owing to the absence of the intercalation/deintercalation process in the former one. This needs more energy and a longer distance for ions to move back to the electrolyte. *P* value at the 1st cycle was found to be 1941 W/kg and minimized to 1633 W/kg at the 150th cycle. Yassine et al. [92], mentioned that the power density is largely linked to internal resistance; therefore, the *P* pattern will be opposed to the ESR pattern. An increase in the ESR value leads to a drop in the value of *P*. After the 175th cycle, the *P* pattern remained almost unchanged with an average value of 1642 W/kg.

## 4. Conclusions

In conclusion, structural, impedance, ion transport parameters, and device performances of the prepared electrolyte have been examined. The transport parameters and DC conductivity estimated from the EIS study reveals the suitability of the electrolyte for device fabrication. The FTIR results and their significant deconvolution were used to detect the interaction that occurs among polymer electrolyte components and determine the transport parameters. The transport properties completed from FTIR analysis is close enough to that achieved from EIS data. The dominant role of ion to the total conductivity was proven from transference number measurement (TNM) as the ionic was found to be 0.93. CS-MC-NH_4_NO_3_–glycerol electrolyte was potentially stable up to 1.87 V. The EDLC was tested for 500 complete charge–discharge cycles. The specific capacitance has a pattern that is similar to that of the energy density as both of them are quite related and the values are stabilized beyond 175th cycles. The specific capacitance and energy density of the EDLC at the first cycle are 50.2 F/g and 6.97 Wh/kg, respectively. Meanwhile, the internal resistance and power density of the EDLC have a similar trend. The average power density and internal resistance of the EDLC throughout the 500 cycles are 1642 W/kg and 63.14 ohms, respectively. The performance of the EDLC can be improved by other researchers via the modifications of electrodes and electrolytes.

## Figures and Tables

**Table 1:** Room temperature ionic conductivity and circuit elements for the plasticized polymer electrolytes (PPEs).

Electrolyte	*R_b_* (Ohm)	Conductivity (S cm^−1^)
CMNG1	200	6.97 × 10^−5^
CMNG2	150	1.16 × 10^−4^
CMNG3	140	1.31 × 10^−4^

**Table 2:** The values of *D*, *µ*, and *n* at room temperature.

Electrolyte	*n* (cm^−3^)	*μ* (cm^2^V^−1^s^−1^)	*D* (cm^2^s^−1^)	*p* (rad)	CPE * (F)
CMNG1	2.02 × 10^21^	2.15 × 10^−7^	5.53 × 10^−9^	1.20	2.29 × 10^−6^
CMNG2	2.73 × 10^21^	2.65 × 10^−7^	6.81 × 10^−9^	1.17	4.35 × 10^−6^
CMNG3	3.02 × 10^21^	2.71 × 10^−7^	6.97 × 10^−9^	1.13	8.00 × 10^−6^

* CPE: constant phase element.

**Table 3:** Transport parameters values of the electrolytes.

Electrolyte	*n* (cm^−3^)	*μ* (cm^2^V^−1^s^−1^)	*D* (cm^2^s^−1^)
CMNG1	4.90 × 10^22^	1.02 × 10^−8^	2.67 × 10^−10^
CMNG2	6.31 × 10^22^	1.11 × 10^−8^	2.87 × 10^−10^
CMNG3	6.80 × 10^22^	1.14 × 10^−8^	2.98 × 10^−10^

**Table 4:** Various types of electrochemical double-layer capacitor (EDLC) with their relative electrodes and specific capacitance value.

System	Active Materials	*C* (F/g)	Reference
hydroxylethyl cellulose + MgTf_2_ + EMIMT + silica nanoparticles	Activated carbon	25.1	[1]
PVA + CH_3_COONH_4_ + BmImCl	Activated carbon	31.3	[86]
MC + NH_4_NO_3_ + PEG	PEG/Activated carbon	38.0	[87]
PVA/polystyrene	Carbon	40.0	[88]
Cellulose + Na_2_SO_4_	Cellulose nanofiber + graphite	43.0	[89]
**CS + MC+ NH_4_NO_3_ + glycerol**	**Activated carbon**	**50.2**	**Present work**

## Data Availability

Data will be made available at request.
